# Ethical implications of HIV self-testing: the game is far from being over

**DOI:** 10.11604/pamj.2016.25.114.8303

**Published:** 2016-10-26

**Authors:** Luchuo Engelbert Bain, Chobufo Muchi Ditah, Paschal Kum Awah, Nkoke Clovis Ekukwe

**Affiliations:** 1 Department of Military Health, Ministry of Defense, Yaoundé, Cameroon; 2 Center for Population Studies and Health Promotion, CPSHP, Yaoundé, Cameroon; 3 Braun School of Public Health and Community Medicine, Hebrew University of Jerusalem, Israel; 4 Faculty of Arts, Letters and Social Sciences, FALSS, Department of Anthropology, University of Yaounde I, Cameroon; 5 Faculty of Medicine and Biomedical Sciences, Department of Internal Medicine and Specialties, University of Yaounde I, Cameroon

**Keywords:** Ethics, Self-testing, HIV, AIDS

## Abstract

The use of combined Anti-Retroviral Therapy (cART) has been revolutionary in the history of the fight against HIV-AIDS, with remarkable reductions in HIV associated morbidity and mortality. Knowing one's HIV status early, not only increases chances of early initiation of effective, affordable and available treatment, but has lately been associated with an important potential to reduce disease transmission. A public health priority lately has been to lay emphasis on early and wide spread HIV screening. With many countries having already in the market over the counter self-testing kits, the ethical question whether self-testing in HIV with such kits is acceptable remains unanswered. Many Western authors have been firm on the fact that this approach enhances patient autonomy and is ethically grounded. We argue that the notion of patient autonomy as proposed by most ethicists assumes perfect understanding of information around HIV, neglects HIV associated stigma as well as proper identification of risky situations that warrant an HIV test. Putting traditional clinic based HIV screening practice into the shadows might be too early, especially for developing countries and potentially very dangerous. Encouraging self-testing as a measure to accompany clinic based testing in our opinion stands as main precondition for public health to invest in HIV self-testing. We agree with most authors that hard to reach risky groups like men and Men Who Have Sex with Men (MSM) are easily reached with the self-testing approach. However, linking self-testers to the medical services they need remains a key challenge, and an understudied indispensable obstacle in making this approach to obtain its desired goals.

## Commentary

Knowing one's HIV serostatus remains a critical and enviable objective public health seeks to attain to guaranteeits early diagnosis, treatment and prevention [1]. Youngs and Hooper in their recent paper in the BMJ Medical Ethics have been firm on the fact that self - testing in HIV stands almost indisputably justified on practical ethical grounds with the main argument being promotion of patient autonomy [2]. Makusha et al, and Frith have also concluded that it is time to give patients the autonomy to decide on whether to use self-testing or not [3, 4]. Frith is firm on the fact that HIV self-testing is truly the ideal with regards to respect of patient autonomy [4]. Autonomy is one of the ethical principles that is continually subject to serious debate and controversy in the bioethics space. Though denied by the very brains behind principalism, Beauchamp and Childress [5], it is and has repeatedly been considered to have greater authority over the other ethical principles (beneficence, non-maleficence and justice). We however think the authors' views have not been holistic enough especially as concerns the global targets to fight against the HIV pandemic. Allowing patients to take decisions with regards to their health as they want is acceptable. However, doing this knowing that even the very educated patients might not always fully understand some of the decisions they do take when they are left to themselves could be a deception of medicine to the very duty it seeks to fulfil, which is enhancing patient wellbeing. Challenging the ideas and perceptions of patients, and addressing their concerns through a frank and acceptable clinical discussion remains the only way, in our opinion, to permit patients to take informed decisions with respect to their health. Informed decision making constitutes the “moral - ethical” backing of true patient autonomy in clinical medicine. Self-testing in HIV as of now does not meet enough this standard and using autonomy as main argument to justify this testing approach can be grossly erroneous. Patients might fail to understand the implications of test results misinterpret some results if not properly trained to do so and even fail to properly identify specific risks that compel an HIV test. Knowing test results without a good appropriate linkage to care apparatus might make investing on self-testing at some point worthless [3, 4, 6].

We agree with Youngs and Hoopers [2] on the fact that there is insufficient evidence to refuse their use since the harms they cause as of now cannot be very much appreciated. It is however reasonable, because the very harms are difficult to ascertain, to implement this practice with lots of caution. There are also limited data however to underestimate the possible harms of this testing approach. It might be interesting to note that with recent reports indicating the high acceptability of this practice [7, 8], a superficial dramatic rehearsal of possible future consequences could be counterproductive in the fight against HIV. Despite the significant progress made to destigmatize HIV -AIDS, it remains a stigmatizing condition in both developed and developing countries [1]. Dealing with HIV in developed countries as an almost stigma free condition as presented by the authors is not only dangerous, but also erroneous [1]. We argue that likening an HIV test to a pregnancy test as used by Youngs and Hooper, cannot only be misleading on the psychosocial appraisal and interpretation arms, but is misleading when discussed as similar entities in clinical ethics. First, pregnancy is not a disease, requires no chronic medication and is far from carrying the stigma that is usually associated to HIV. For patients who test positive and are put on treatment, compliance to recommended combined AntiRetroviral Therapy (cART) remains key to ensure success in reducing not only HIV associated morbidity and mortality, but also transmission of this infection [1, 9]. With glaring linkage to care gaps associated with self-testing as of now, overreliance on this approach might therefore be counterproductive. We agree with Paltiel and Walensky [6], who propose that self-testing as of now is reasonably not a substitute for standard screening in health care facilities. Developed countries with reasonably high levels of education in the general population still deserve to pay great attention to the HIV transmission dynamics especially amongst the high risk groups. High risk groups like MSM, people with lower levels of education and today growing numbers of illegal immigrants cannot be left or encouraged entirely to self-test in the context of HIV. They are far from being considered insignificant in the UK. In a mathematical model in the Unites States of America (U.S.A), Katz et al reported that replacing hospital based screening with home based screening amongst Men Who Have Sex with Men (MSM) in Seattle may increase the prevalence of HIV in this high risk group [7]. It is also unclear however if patients will always appropriately identify risky situations to self-screen for HIV, and if they will behave appropriately after getting the test results. Self testing in Sub-Saharan African countries deserves a deeper review and greater caution. Not only might most patients not fully understand how to use the tests, interpret results, identify risky situations to get the test done, linkage to care could be inadequately acted upon. Regions in some these countries may not have the means to take care of all persons that could potentially test positive.

We agree with Youngs and Hoopers [2] on the fact that self-testing has the potential to increase numbers of persons tested, even had to reach and high risk groups like men and (MSM) and prevent further transmission [2–4]. This assertion only becomes reasonable when linkage to care after obtaining HIV test result, whether positive or negative is assured [3, 6]. Paltiel and Walensky have argued that most persons that would opt for self-testing could be potentially more refractory to care, most skeptical about the health system and more worried about stigma and privacy issues [6]. Fear, stigma, mistrust in health systems, confidentiality issues and poor quality counseling might discourage clients from seeking care [6, 7, 9]. Wood et al have raised the concern that self-testing in HIV might be a key opportunity lost in diagnosing other Sexually Transmitted Infections. False assurances in case of false negative results, inadequate understanding of the window period implications in case of negative results and missed opportunities of counseling and linkage to care make self-testing not an ideal, as a sole option within the HIV prevention and management package [3, 6, 8].We agree with Youngs and Hooper that self-testing has the potential of increasing the number persons who come to know their HIV status [2]. We propose and respect the ideal of a healthy and trusting clinician-patient relationship, necessary to challenge the knowledge and opinions of patients, to permit them to take informed choices as key, especially when it comes to a “chronic” infectious disease like HIV. Even in a hypothetical situation where everyone self-tests for HIV but remains unlinked to appropriate care, the rationale behind the test, which involves behavioral change and timely initiation of treatment for those who test positive, might be completely missed. Research with regards to linkage to care and its associated costs is still very limited within the context of HIV self-testing. Without ensuringlinkage of self-testers to regular health care facilities as a precondition, the very usefulness of this approach might turn out to be unproductive. Without downplaying on the importance of knowing and on time one's HIV status within the context of HIV prevention and care, we argue that self-testing should simply supplement the current clinic based HIV screening practices [6]. The success of HIV self-testing might strongly depend on the trust and confidence the population has on its health system [6, 9]. Respect of patient autonomy to justify self-testing assumes that the patient's choices are always well informed. This can be grossly erroneous and even potentially dangerous, if the health system allows the general public to deal with, on their own, the enormous amount of information they get under the assumption that the choices are adequately informed. At times easily overlooked, the most critical aspect of an HIV test is not knowing the test result, but adopting an appropriate behavior after knowing this result. Testing negative today is no guarantee of being HIV free if the client does not understand fully the meaning of the window period or a false negative result. An inadequate counseling package might also not guarantee compliance to recommended medical care for those who test positive, nor ensure an appropriate behavioral change for those who test negative. Actively improving efforts to link “self-testers” to care in our opinion remains the key, if not the only justification to expand self-testing for HIV. It is too early to welcome self-testing for HIV as a panaceia to overcome HIV screening barriers in current facility based screening settings, especially on grounds of respect of patient autonomy. It could be welcome as a public health measure to accompany traditional clinical setting screening, on grounds that measures to render information on the screening kits understandable are put in place, and measures to link patients to care after the test improved upon.

## References

[1] Maartens G, Celum C, Lewin SR (2014). HIV infection: epidemiology, pathogenesis, treatment, and prevention. Lancet..

[2] Youngs J, Hooper C (2015). Ethical implications of HIV self-testing. J Med Ethics..

[3] Makusha T, Knight L, Taegtmeyer M (2015). HIV self-testing could “revolutionize testing in South Africa, but it has got to be done properly”: perceptions of key stakeholders. PLoS One..

[4] Frith L (2007). HIV self-testing: a time to revise current policy. Lancet..

[5] Ahmadi Nasab Emran S (2015). The four-principle formulation of common morality is at the core of bioethics mediation method. Med Health Care Philos..

[6] Paltiel AD, Walensky RP (2012). Home HIV testing: goodnews but not a gamechanger. Ann Intern Med..

[7] Katz DA, Cassels SL, Stekler JD (2014). Response to the modeling analysis by Katz et al on the impact of replacing clinic-based HIV tests with home testing among men who have sex with men in Seattle: authors' reply. Sex Transm Dis..

[8] Wood BR, Ballenger C, Stekler JD (2014). Arguments for and against HIV self-testing. HIV AIDS (Auckl)..

[9] Eyal N (2014). Using informed consent to save trust. J Med Ethics..

